# Supplementary material for: Assessing modern river sediment discharge to the ocean using satellite gravimetry
Source: Nat Commun. 2018 Aug 23;9:3384. doi: 10.1038/s41467-018-05921-y (PMC6107634; doi:10.1038/s41467-018-05921-y)
Supplement: Supplementary file 1 — Supplementary Information [file 41467_2018_5921_MOESM1_ESM.pdf]

Supplementary Information

Assessing modern river sediment discharge the ocean using satellite gravimetry

Mouyen et al.

SUPPLEMENTARY FIGURES

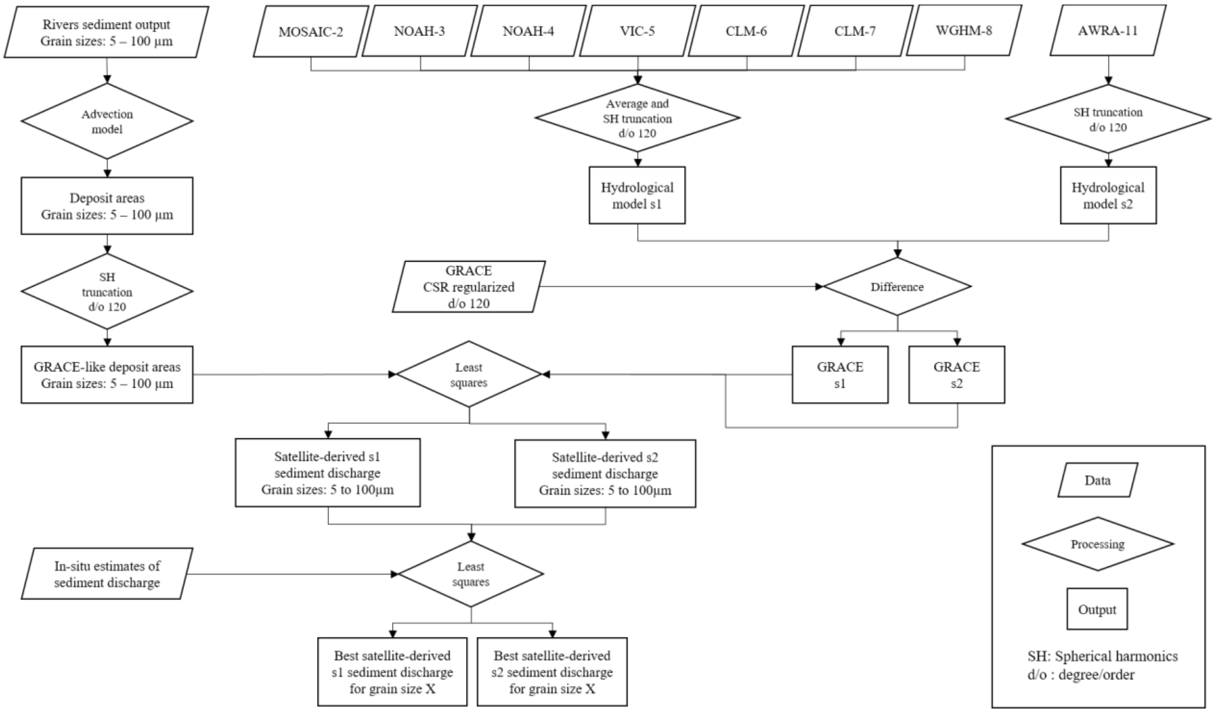

Supplementary Figure 1: Schematic of the general workflow of our analysis. This schematic shows how our sediment advection model and GRACE data, with their hydrological corrections, are combined to retrieve the sediment discharge to the ocean.

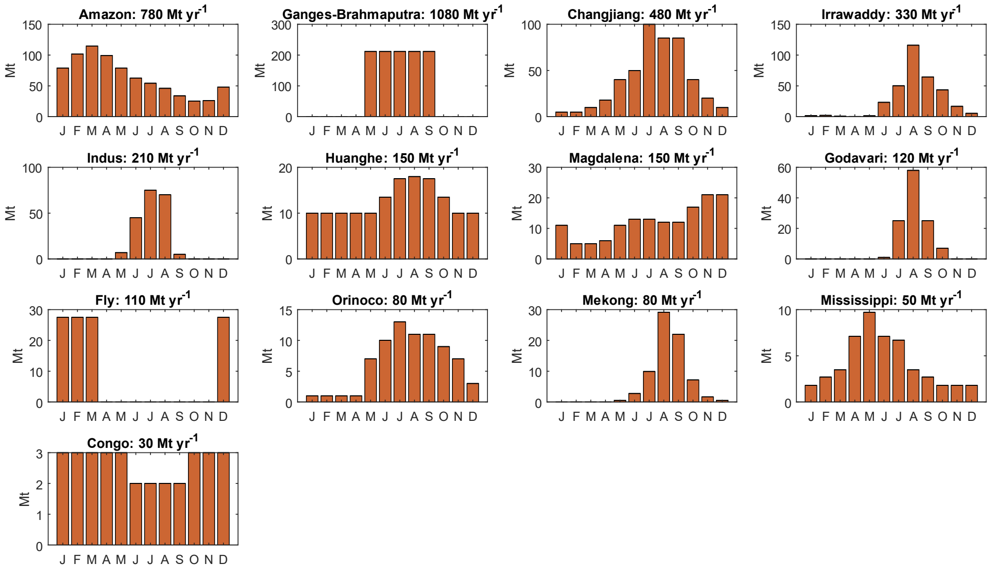

Supplementary Figure 2: Monthly sediment discharge at each of the thirteen rivers used in this study. The total, yearly, sediment discharge is given in each subpanels' title.

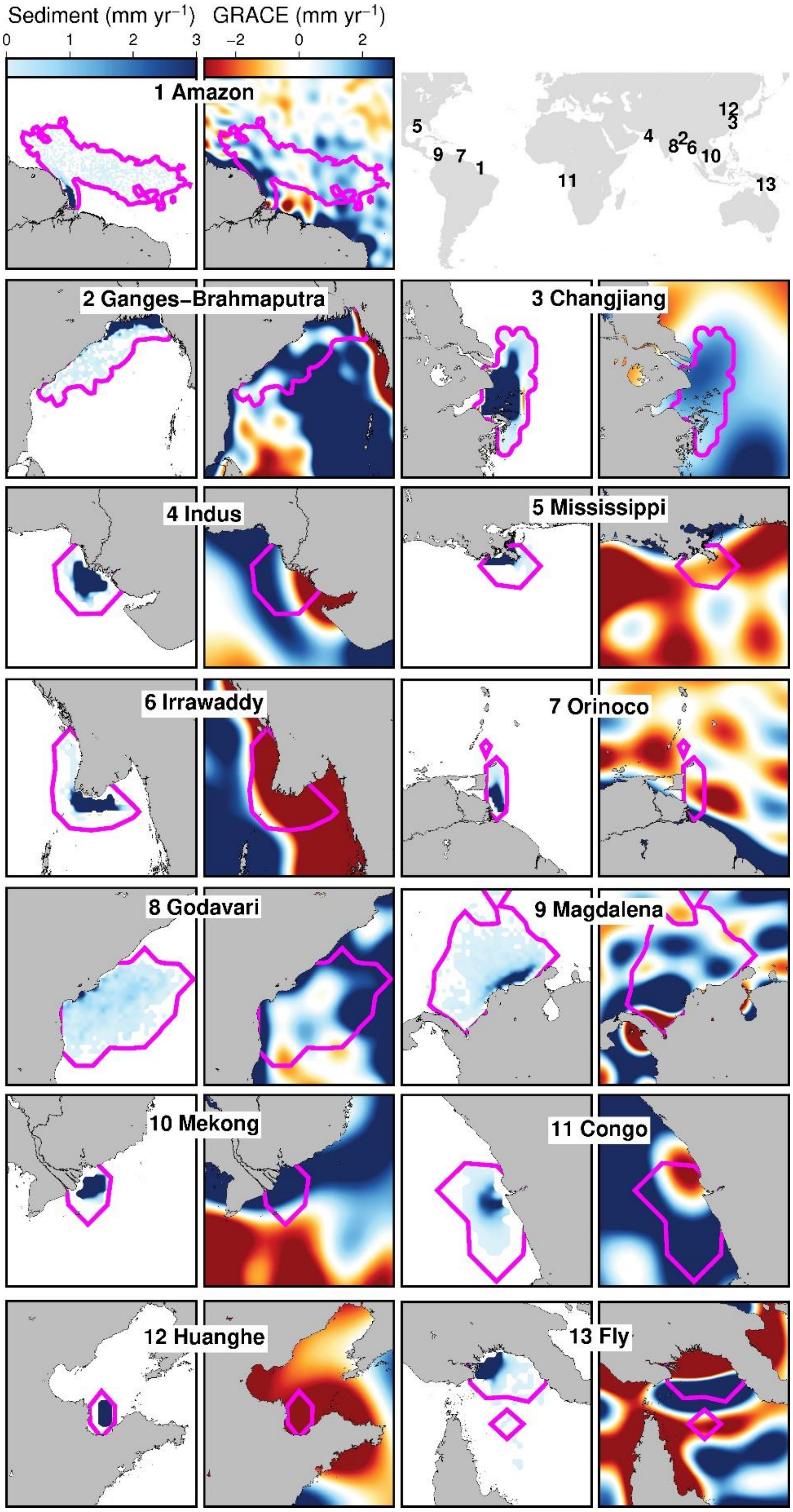

Supplementary Figure 3: Sedimentation zones versus GRACE data. Sedimentation rates (columns 1 and 3) and GRACE equivalent mass rates (columns 2 and 4) over the same areas with the sedimentation area superimposed as a pink contour, for the 13 rivers of this study. Colors are scaled homogeneously for both GRACE and sediment maps. Units are equivalent mm of sediment per year assuming sediment replaces water. The top right world-map locates each river by its number.

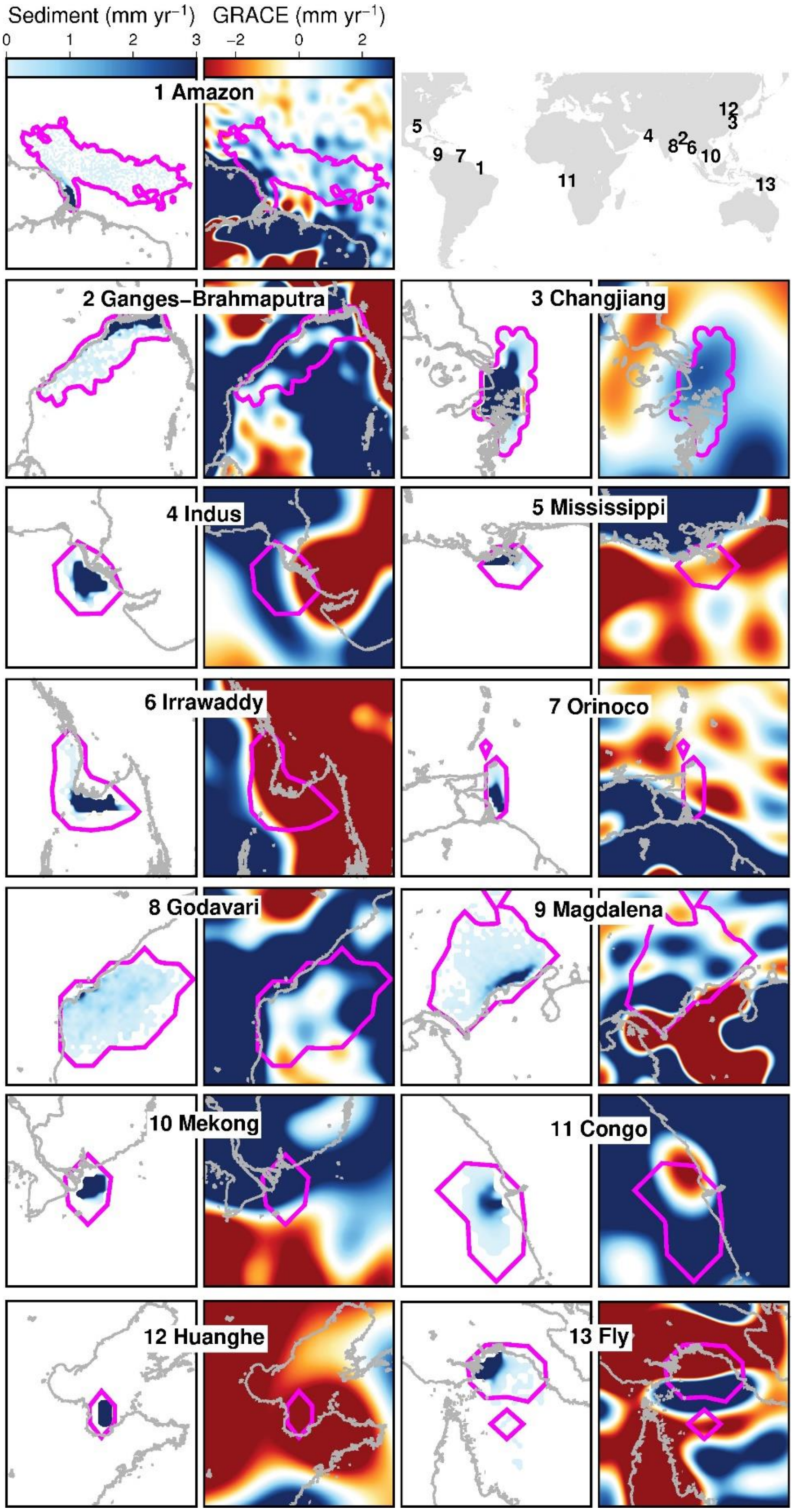

Supplementary Figure 4: Sedimentation zones versus GRACE data. Same as Fig. 1 and Supplementary Fig. 3 but without the mask on the continents to not hinder the GRACE signal. Spatial correlation exists from the land to the ocean, showing possible leakage of the hydrological signal, which cannot be fully corrected (e.g. Indus, Godavari, Mekong). However, some rivers do not exhibit such correlation (e.g. Changjiang, Magdalena). This suggests that the oceanic signal is well separated from the land's influence.

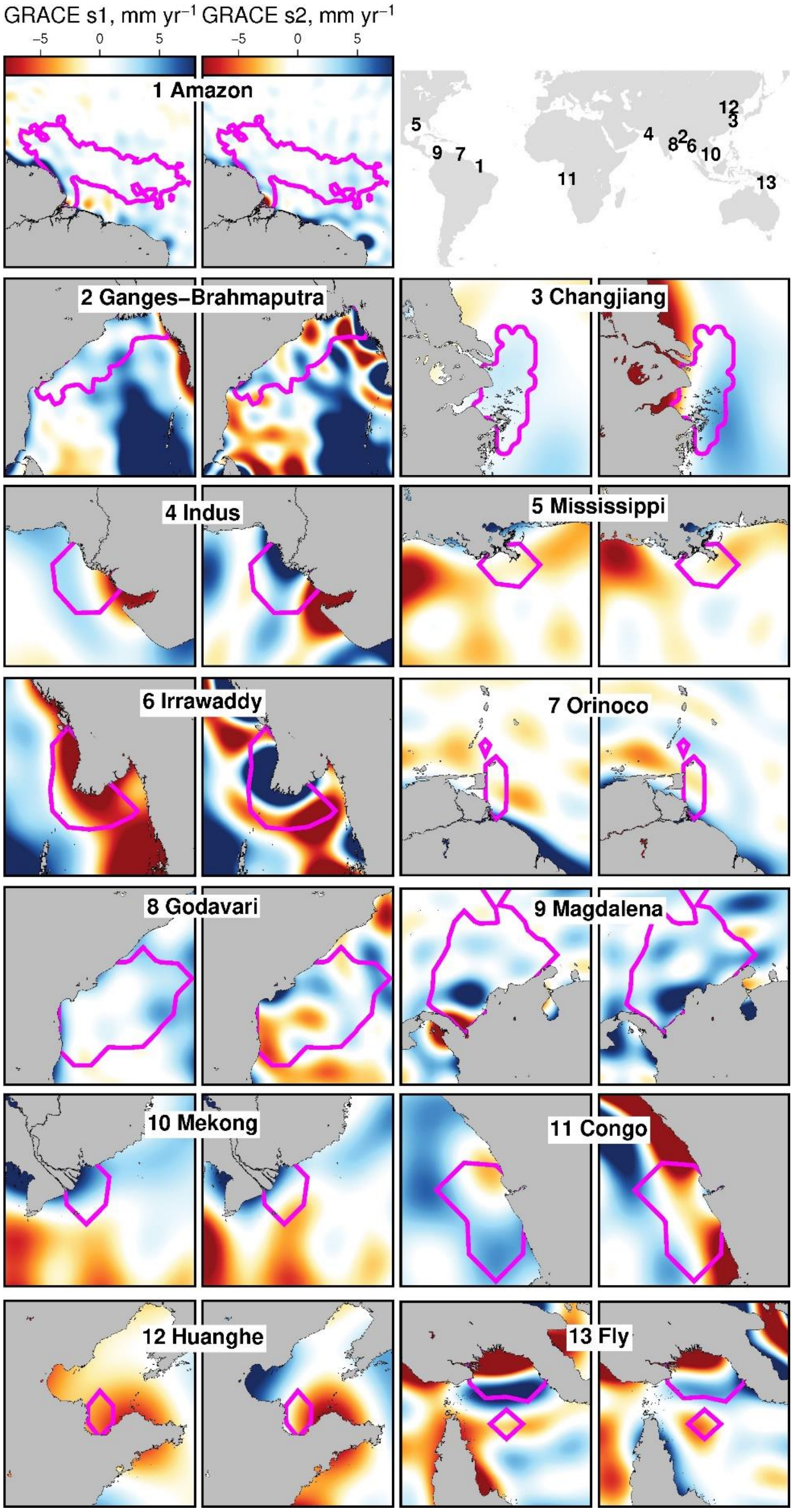

Supplementary Figure 5: Hydrological corrections applied to GRACE data. Comparison of the GRACE s1 (columns 1 and 3) and s2 solutions (columns 2 and 4), which use two different hydrological corrections (see Method). The sedimentation area is modelled using the particle diameter given in each title and superimposed as a pink contour. The GRACE signal is in mm.yr^{-1} of equivalent sediment thickness (assuming sediment replaces water).

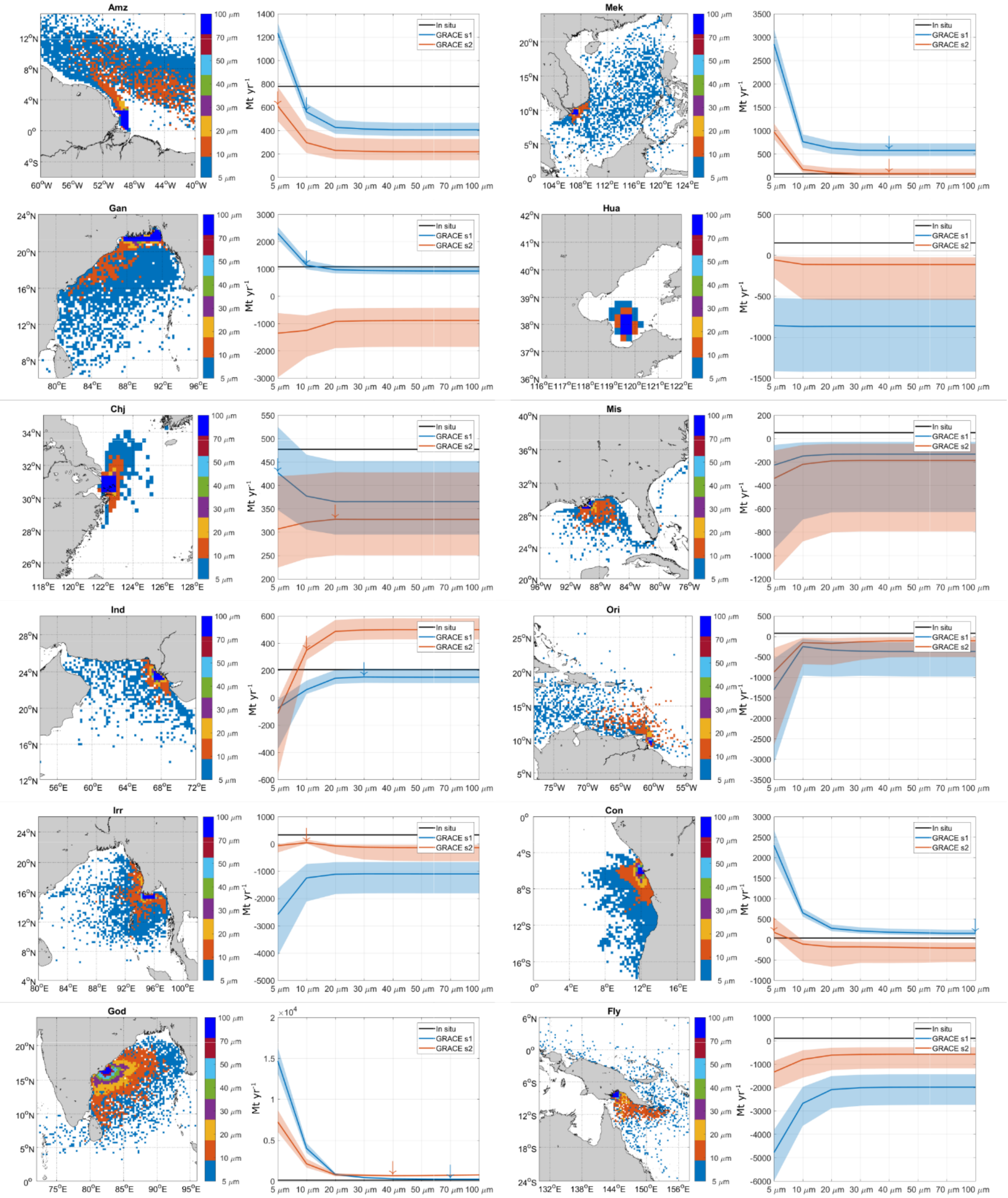

Supplementary Figure 6: Grain size effect on the sedimentation zones. Columns 1 and 3: Spatial extension of the estimated sedimentation zone depending on the particles diameters used in the sediment advection model. Columns 2 and 4: Satellite-derived sediment discharge (SSD) adjusted from GRACE s1 (blue line) and s2 (red line) solutions, as a function of the particles diameters. The reference sediment discharges derived form in situ measurements (independent from the particle diameter) is also plotted in black for comparison. For positive SSD, grain size leading to the smallest difference between the SSD and the reference sediment discharge are pointed with arrows.

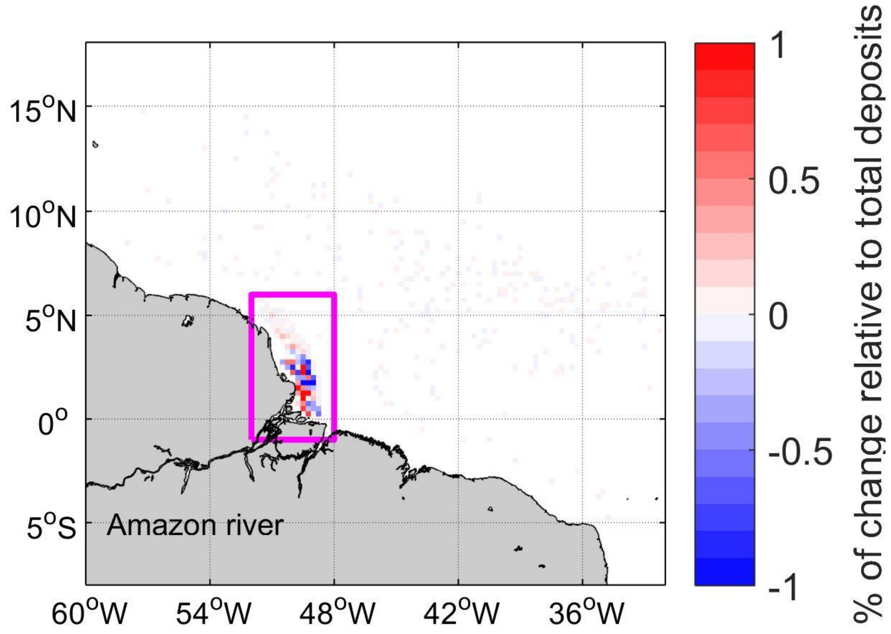

Supplementary Figure 7: Tidal currents effect on sediment advection. Change in the spatial deposition of sediment when tidal currents are added to ECCO2 currents during the simulation of sediment advection. If A is the spatial deposition only considering ECCO2 currents and B the spatial deposition considering both ECCO2 and tidal currents, then this map corresponds to $100(B - A)/A$.

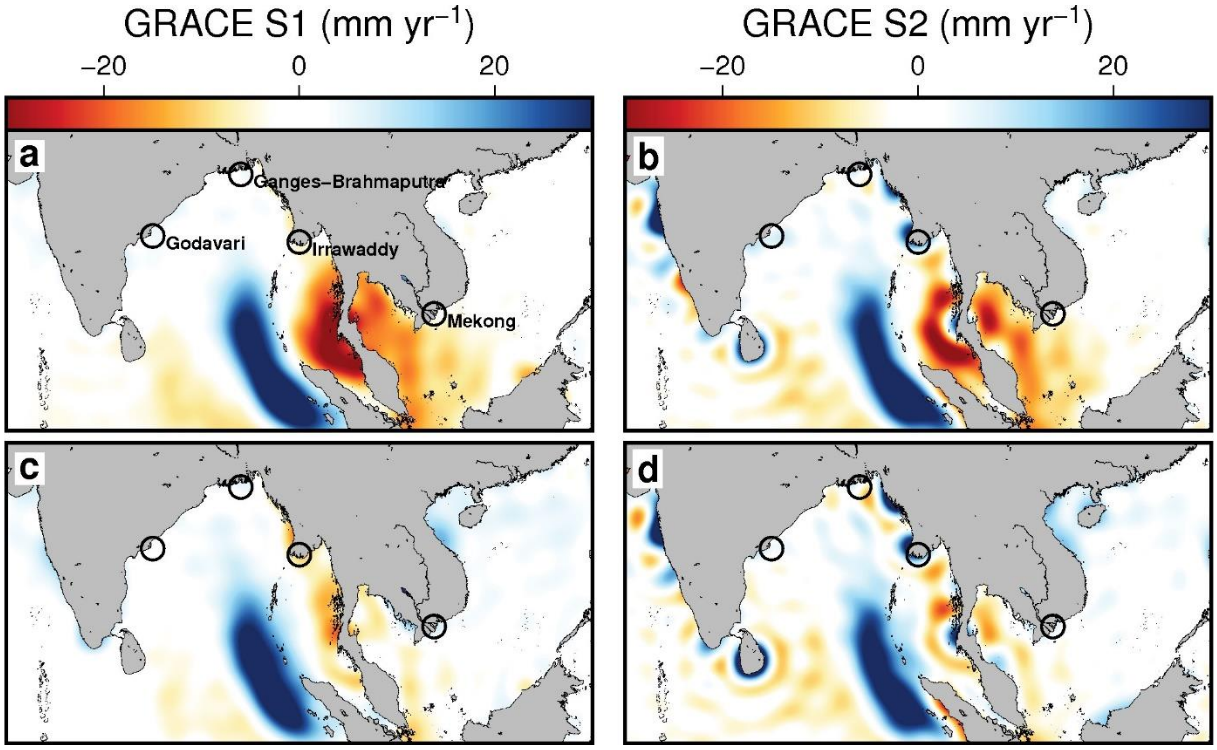

Supplementary Figure 8: Coseismic effect of Sumatra and Nias on GRACE data. Comparison of the GRACE s1 and s2 solutions when trends are computed over the entire length of the GRACE time series, hence including the coseismic effects of Sumatra and Nias earthquakes (a and b), or when trends are computed over GRACE time series starting after these two large earthquakes (c and d). The coseismic effect (a large gravity negative offset) is significantly reduced in the Gulf of Thailand, where the Mekong river arrives. In the Bay of Bengal, even after removing coseismic effects, large positive gravity trends remain in the GRACE trends due to ongoing postseismic mass redistributions. Units are equivalent mm of sediment per year assuming sediment replaces water

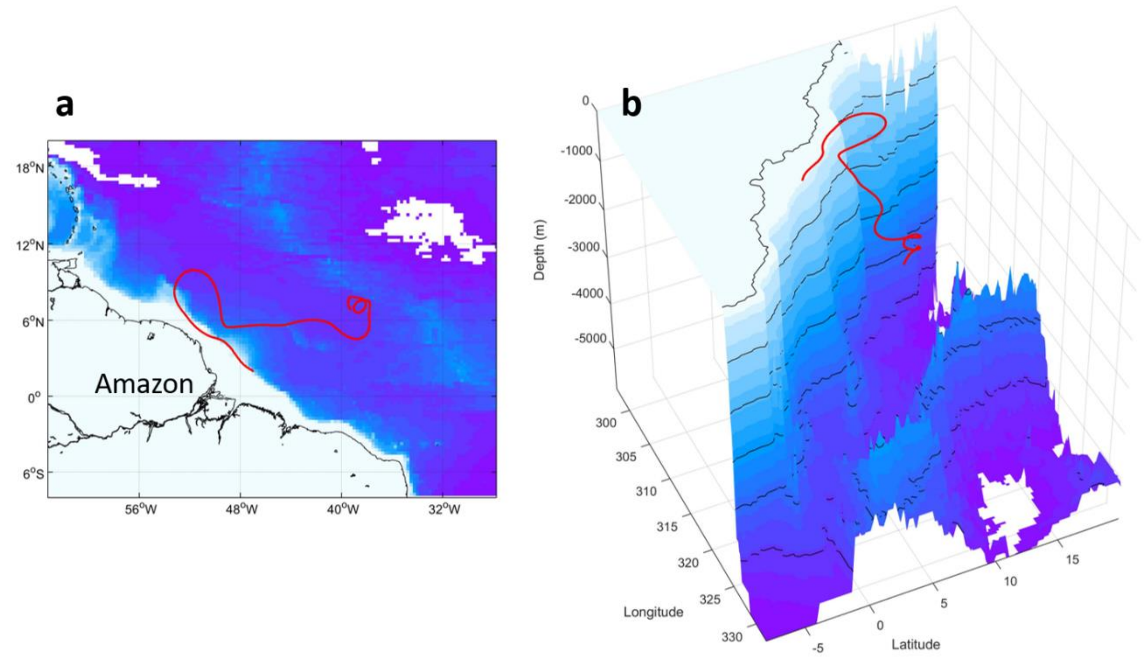

Supplementary Figure 9: Particle advection example. Example of the sinking path of a particle from the Amazon river mouth to the Atlantic Ocean, as computed by our advection model in the ECCO2 oceanic velocity field. a) map view and b) 3D view.

Note	Map: True mass (red outline) [1 Gt], an a-priori as blue surface	True mass @ GRACE resolution	Foward model resolved with the right a-priori [mm]	Residuals considering the right a-priori [mm]
T120	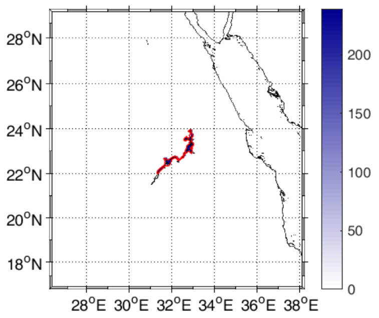	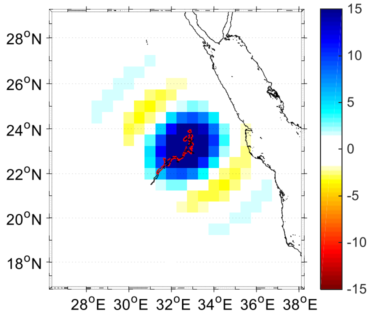	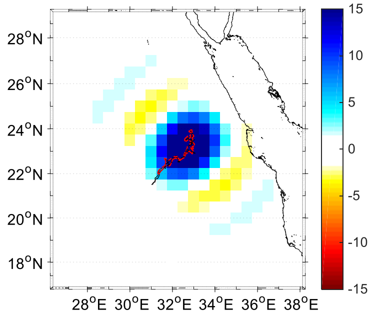	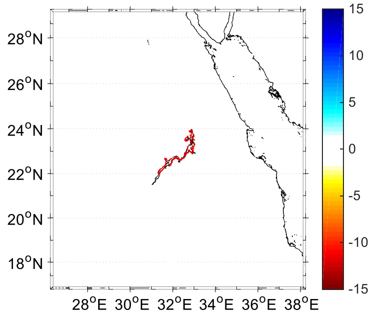
T96G300		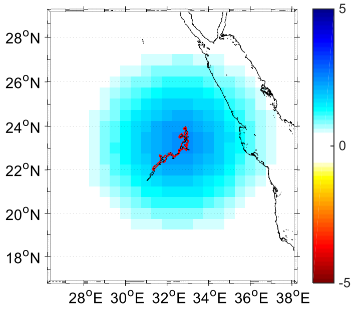	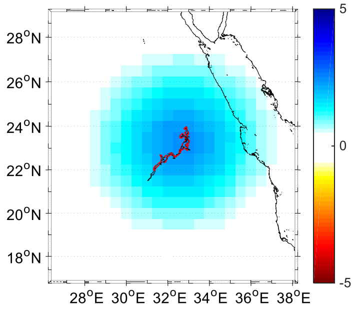	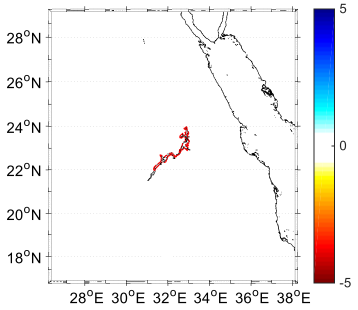
T96DDK7		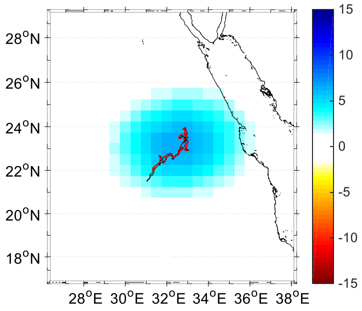	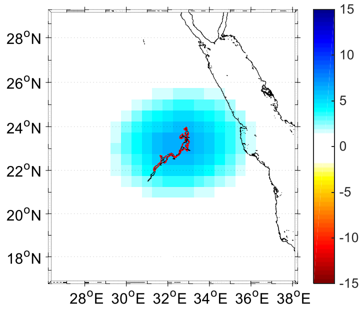	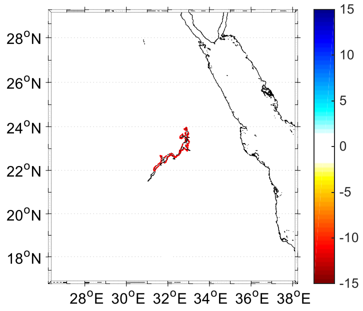

Supplementary Figure 10: Ability to recover a 1 Gt mass based on different processing strategies. T120: truncation at degree 120 with no further filtering for regularized products, T96G300: truncation at degree 96 plus 300-km Gaussian smoother for general GRACE processing, T96DDK7: truncation at degree 96 plus DDK-7 filter as an alternative processing. The left panel shows the a-priori, which is well positioned over the lake area. Next panel on the right is the true mass converted to GRACE resolution, following panel is the forward model resolved based on the right a-priori mass distribution, last panel are the residuals.

Note	Map: Wrong a-priori [1 Gt]	True mass @ GRACE resolution [mm]	Foward model resolved with a wrong a-priori [mm]	Residuals considering a wrong a-priori [mm]
T120	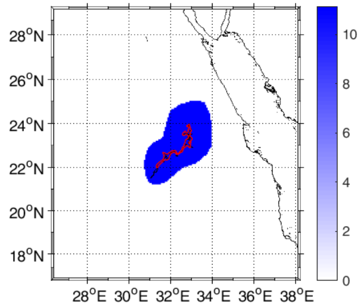	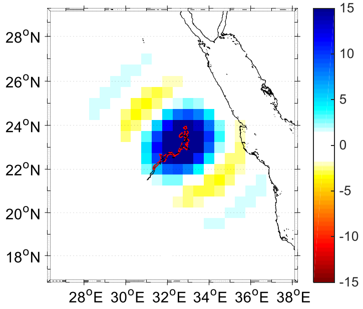	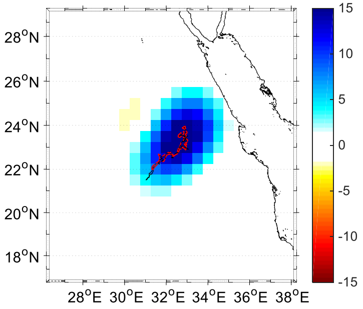	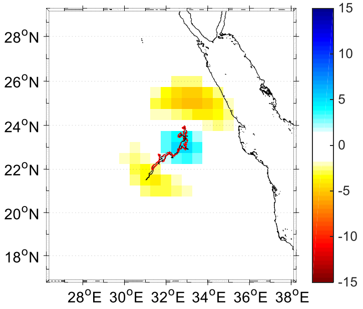
T96G300		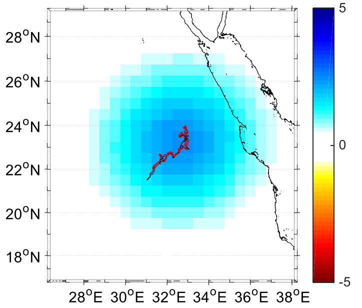	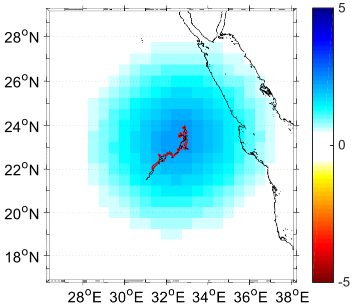	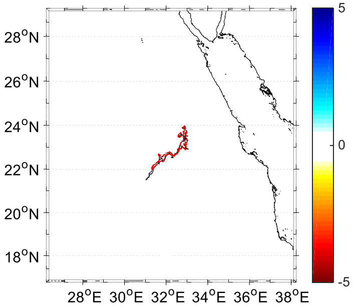
T96DDK7		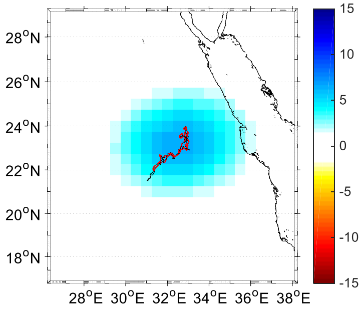	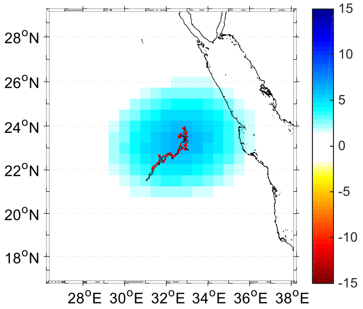	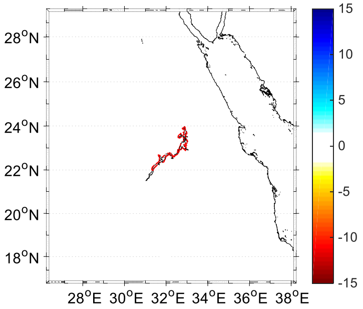

Supplementary Figure 11: Ability to recover a 1 Gt mass based on different processing strategies. Same as Supplementary Fig. 10 but a wrong a-priori mass distribution is considered. We consider the center of mass to be well known, but the area not constrained.

Note	Information Map : Noise map [mm]	True mass+noise @ GRACE resolution [mm]	Foward model resolved with the right a-priori [mm]	Residuals considering the right a-priori but including noise [mm]
T120	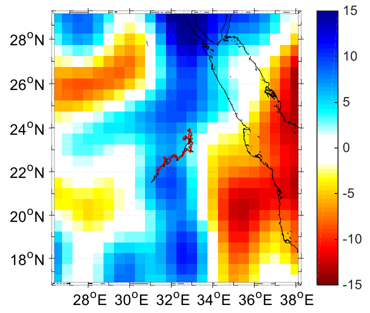	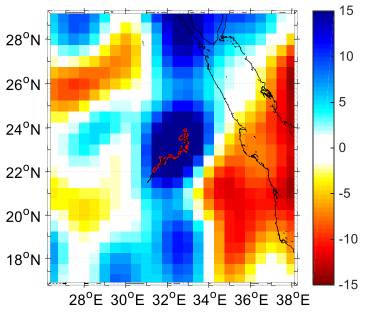	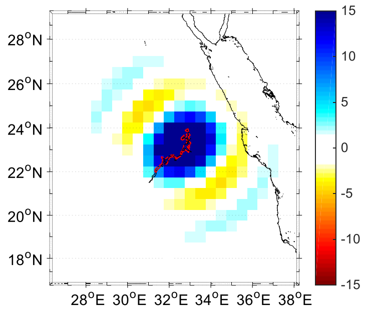	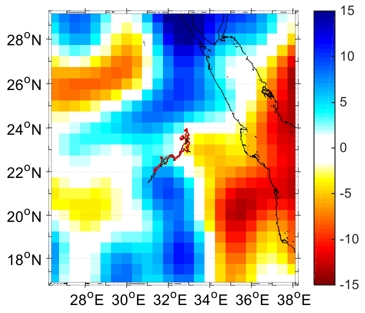
T96G300	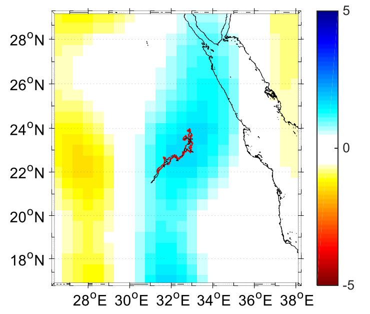	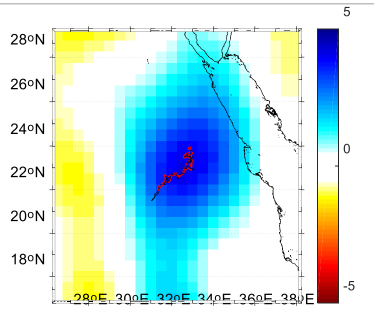	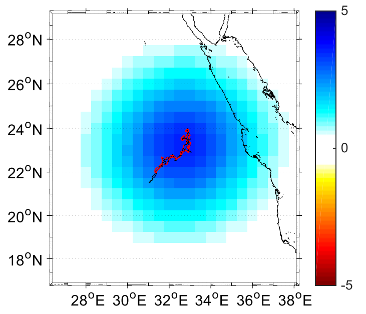	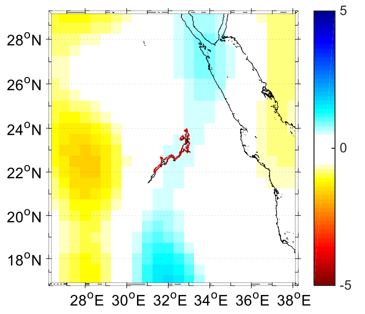
T96DDK7	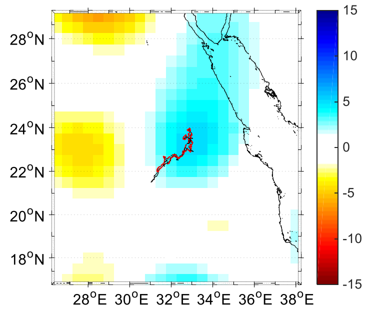	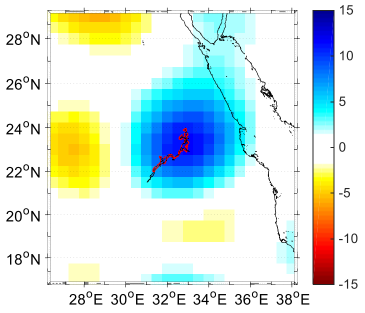	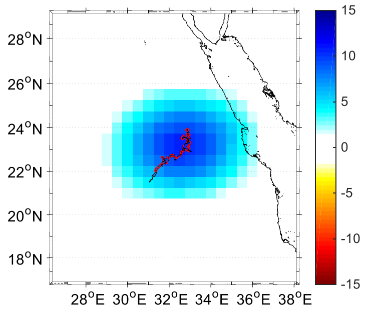	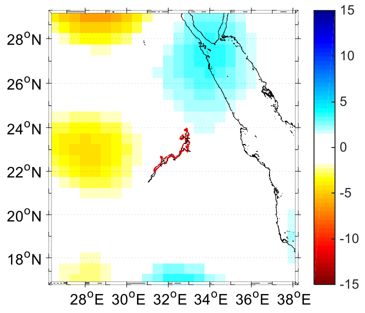

Supplementary Figure 12: Ability to recover a 1 Gt mass based on different processing strategies. Same as Supplementary Fig. 10, with a right a-priori mass distribution but with noise added.

Note	Information Map : Noise map [mm]	True mass+noise @ GRACE resolution [mm]	Foward model resolved with the wrong a-priori [mm]	Residuals considering the wrong a-priori and including noise [mm]
T120	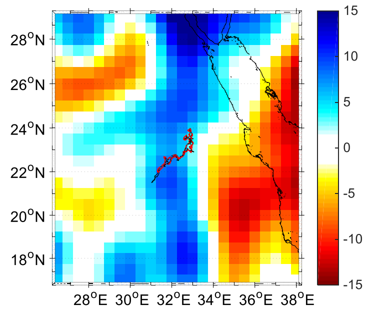	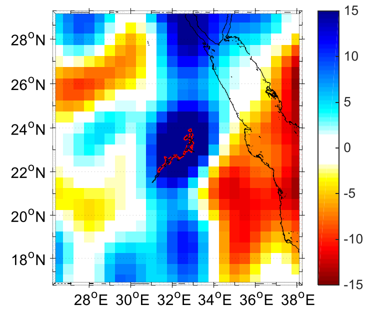	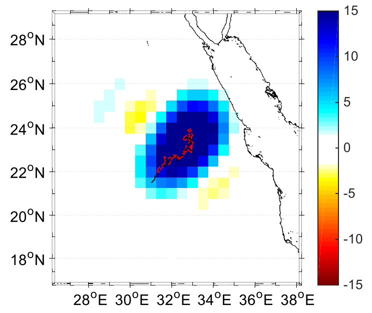	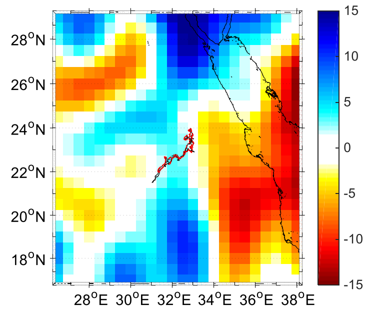
T96G300	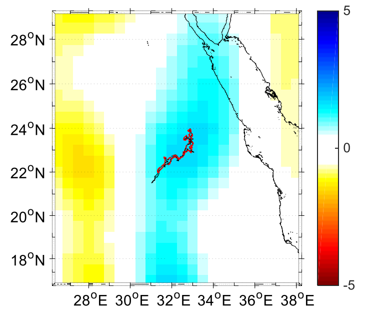	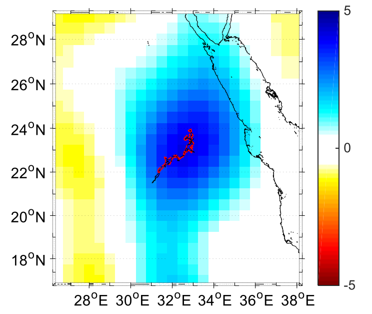	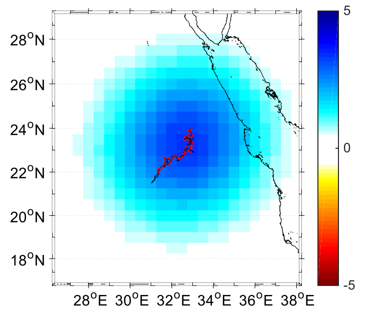	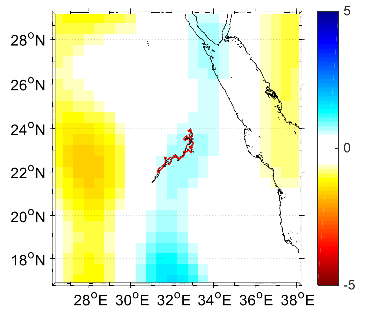
T96DDK7	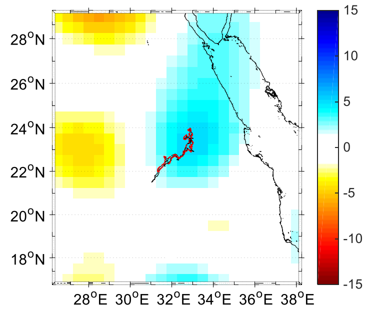	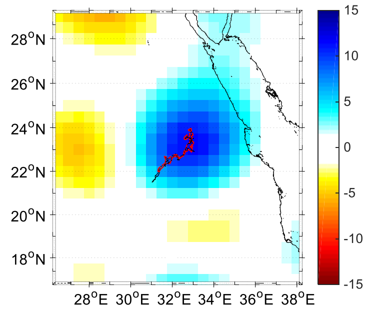	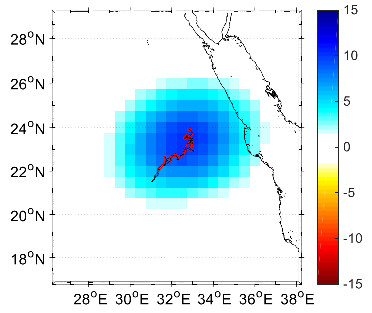	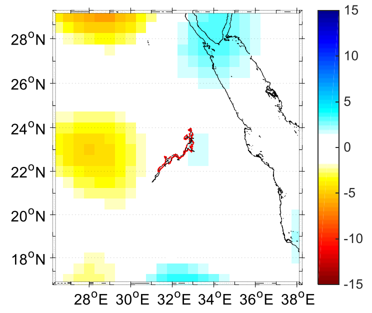

Supplementary Figure 13: Ability to recover a 1 Gt mass based on different processing strategies. Same as Supplementary Fig. 10 with both a wrong a-priori position and noise.

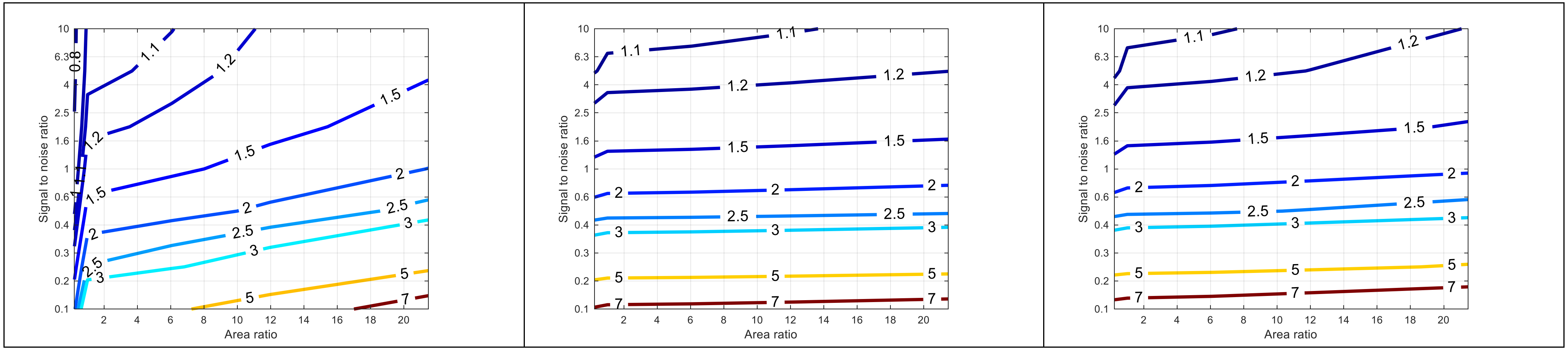

Supplementary Figure 14: Synthesis of the numerical experiments shown in Supplementary Figures 10 to 13. Mass overestimation factor for different signal-to-noise ratio (0.1 to 10), area ratio (0.1 to 20), for each processing strategy (from left to right, T120, T96G300, T96DDK7).

Processing strategy	GRACE data [mm yr^{-1}]	Forward model for lake Aswan alone [mm yr^{-1}]	Residuals [mm yr^{-1}]
T120	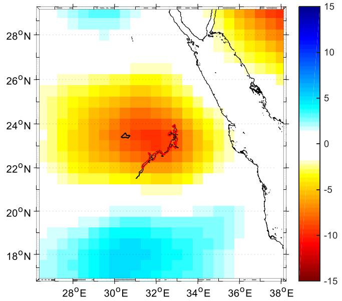	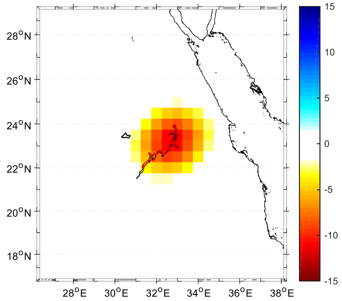	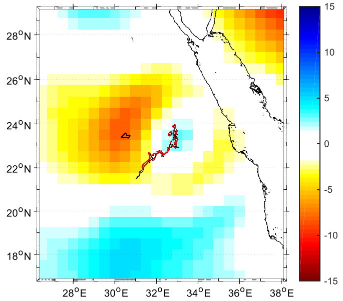
T96G300	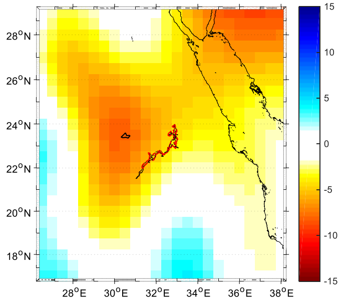	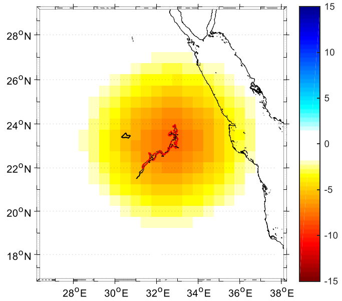	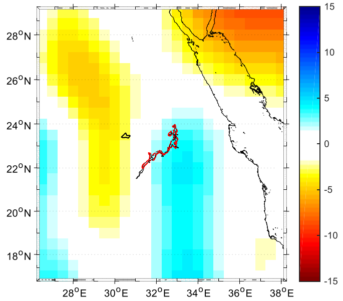
T96DDK7	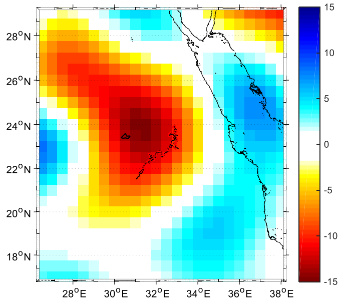	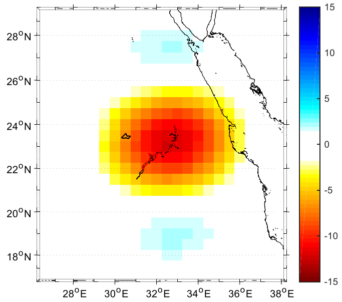	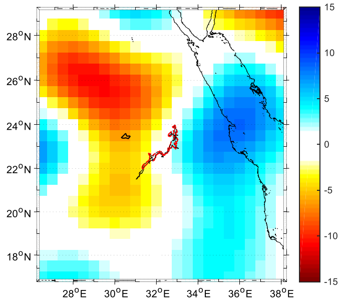

Supplementary Figure 15: Estimation of Aswan lake long-term mass changes. Lake Aswan is shown as the red outline, lake Toshka is also outlined in black, on the West of the Aswan lake. As the signal-to-noise ratio is ~0.5, mass changes in the lake Aswan explains a limited part of the original GRACE map, leaving large residuals in no specific spatial structure for the 2 low resolution products. For regularized data, though, the potential impact of Lake Toshka is clearly visible.

Processing strategy	GRACE data [mm yr^{-1}]	Forward model for lake Aswan and Toshka together [mm yr^{-1}]	Residuals [mm yr^{-1}]
T120	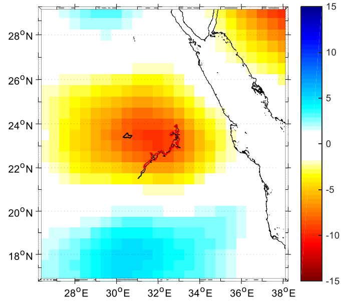	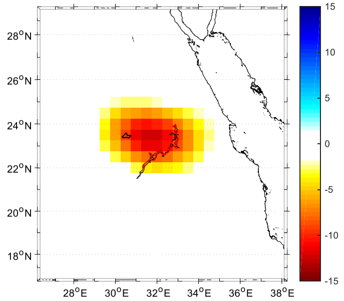	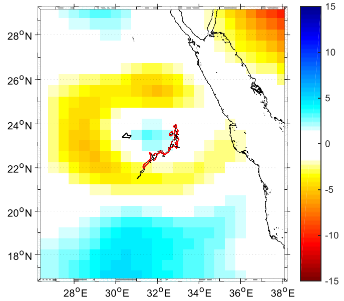
T96G300	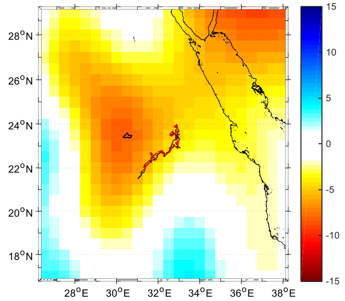	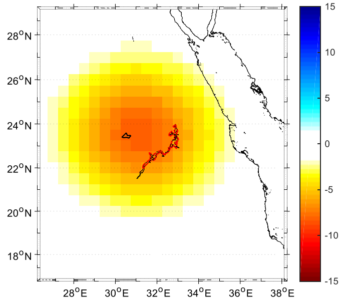	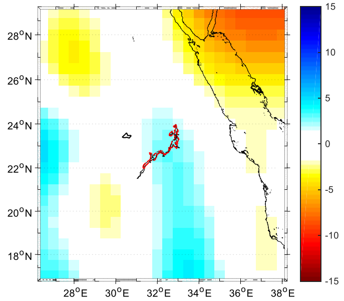
T96DDK7	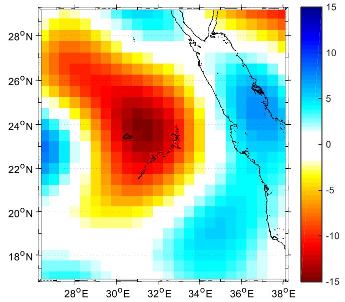	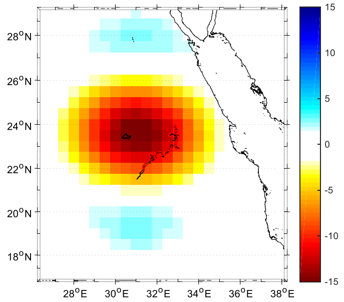	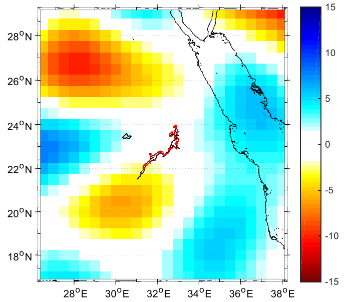

Supplementary Figure 16: Estimation of Aswan and Toshka lakes long-term mass changes. Lake Aswan is shown as the red outline, lake Toshka is also outlined in black, on the West of the Aswan lake.

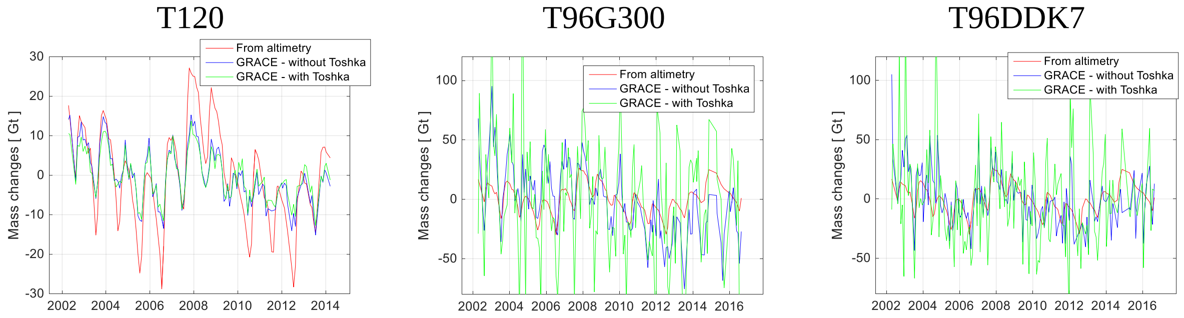

Supplementary Figure 17: Time variability of the mass changes. Inverted mass changes time series on the Aswan lake for the 3 GRACE solutions and compared to mass changes estimated by satellite altimetry.

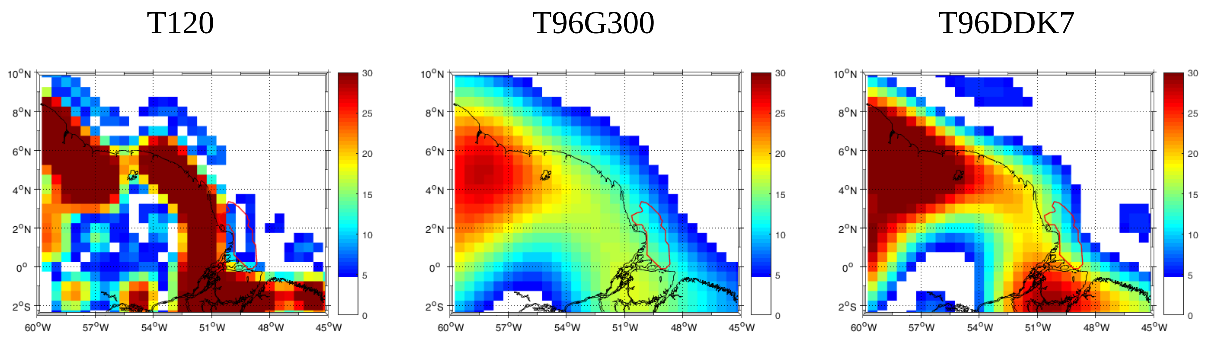

Supplementary Figure 18: Uncertainties generated by continental water storage changes. Standard deviation among LSM trends [mm yr^{-1}] for different processing strategies. The main sedimentary deposition center for the Amazon basin is outlined in red.

SUPPLEMENTARY TABLES

River	Time	Information
Amazon	1995-2007	Surface suspended sediment sampling and remote sensing (MODIS) data, monthly averaged[1].
Ganges-Brahmaputra		Most of the sediment export occurs during the monsoon season. Therefore, we equally distribute the yearly sediment export from May to September[2].
Changjiang	1950-1980	Suspended sediment concentration at the Datong station (downstream, lower Changjiang). Although present yearly sediment discharge might be lower than before (152 Mt yr^{-1} over 2003-2010[5]), we assume that the monthly pattern of sediment discharge is identical[3,4].
Irrawaddy	1877-1878<br>2005-2006	Concentrations are based on an average of surface, middle, and near-bottom depth suspended sediment sample measurements[6].
Indus	1960-1998	Upper Indus. The sediment discharge is computed from river water discharge and suspended sediment rating curves. We use the monthly mean sediment yield computed at station Besham Qila (rating curve regression coefficient $r^2 = 0.8$)[7].
Huanghe	2000-2006	35% of suspended sediment is delivered to the sea during the flood season (July–September). Thus, we share the yearly budget of sediment discharge (150 Mt yr^{-1}) with 35% equally distributed from July to September and 65 % over the other months[8,9].
Magdalena	1975-1995	Monthly mean sediment discharge estimates downstream (Calamar station) are based on the measured sediment concentrations, cross-multiplied with water discharge[10].
Godavari	1970-2009	Average monthly sediment discharges at Polavaram gauging station, 41 km upstream the delta apex[11].
Fly	1992: 8 weeks and<br>April to June 1995	"The net sediment export from the distributary channels during the SE Trade season is zero and suggest that sediment may be exported during the monsoon season."<br>With:<br>- SE Trade season: April to November<br>- Monsoon season: December to March<br>Thus, we homogeneously distribute the yearly sediment export over the 4 monsoon months, December to March[12,13].
Orinoco	2007-2010	Total suspended sediment using data collected at the Ciudad Bolivar stream gauging station. Surface water samples were collected from the centre of the river channel at 10-day intervals[14].
Mekong	1962-2002	Monthly averaged total suspended sediment measured using sample collected at 0.3 m below the water surface in the middle of the river at Pakse station, in the lower Mekong[15,16].
Mississippi	2008	We use the total suspended sediment discharge in the Mississippi River at Belle Chasse, Louisiana as measured by calculated ratings curve[17].
Congo	2006-2010	Total suspended sediment computed from samples that were collected at monthly intervals from 30 cm below the water surface in the middle of the channel cross-section between Brazzaville and Kinshasa where suspended sediment is homogeneously distributed[14].

Supplementary Table 1: River discharge in situ data. Reference, period of time and sediment discharge information used for each studied river in order to build Supplementary Fig. 2. The main information for modelling the sedimentation zones is the time variability of the mean monthly sediment discharge rather than the absolute mass of sediment delivered. Indeed, the sediment advection model is used to constraint the spatial distribution of the sedimentation, which depends on the dynamics of both the sediment discharge and the oceanic currents. The accumulated mass of sediment is inverted from GRACE and only compared to in-situ sediment discharge measurements. Therefore, we prefer data providing monthly sediment discharge patterns rather than yearly-averaged sediment discharge. When the references we use do not span the time period of the GRACE measurements, we assume that, although the total yearly sediment discharge can change across time, the time-variability of the discharge remains identical.

River	In situ<br><br>*Mt yr[-1]*	SSD (GRACE trends computed from 2002 to 2012)<br><br>*Mt yr[-1]*	SSD (GRACE trends computed from June 2005 to 2012, no Sumatra and Nias coseismic effects)<br>*Mt yr[-1]*
Ganges-Brahmaputra	1081	1200±190<br>40±210	1140±200<br>-890±750
Irrawaddy	333	-720±440<br>220±400	-1100±610<br>50±270
Godavari	119	560±80<br>1320±80	230±90<br>650±120
Mekong	75	-580±520<br>-730±610	580±150<br>80±110

Supplementary Table 2: Effect of Sumatra-Nias coseismic gravity change on satellite-derived sediment discharge (SSD). Consequence of using GRACE trends computed over the entire 2002-2012 time series (hence spanning the coseismic effects of Sumatra and Nias earthquakes) on the SSD computation, for both s1 and s2 GRACE solutions (blue and red fonts, respectively), for rivers located near the epicenters of Sumatra and Nias earthquakes. Using trends computed after theses earthquakes occurred (starting in June 2005) gives more reasonable values at the Mekong and Godavari rivers. However, there is no improvement for the Ganges-Brahmaputra and the Irrawaddy because of significant postseismic effects, which still alter the gravity trends in their regions.

**Description**	ECCO2 combines a general circulation model (GCM) of the ocean with satellite and in-situ data to provide a "quantitative depiction of the time-evolving global ocean state" [18]
**GCM**	MITgcm, based on Navier Stokes equations (*45*, *46*) in a volume-conserving configuration and including<br>• a global full depth ocean (*47*, *48*)<br>• a sea-ice model (*49*)
**Data assimilated**	Altimetry, gravity, drifter, hydrography, sea-ice observations
**Spatial resolution**	• Horizontal: 18 km on average<br>• Vertical: 50 levels of depths with thicknesses ranging from 10 m at the surface to 450 m at maximum depth (6150 m)
**Time resolution**	3 days
**Diagnostic variables**	Surface fluxes, sea surface height, bottom pressure, mixed and mixing layer depths, sea-ice thickness, concentration, salinity, ocean temperature, density, velocity and eddy transports of mass, temperature, and salt.
**Data access**	ftp://ecco2.jpl.nasa.gov/data1/cube/cube92/lat_lon/quart_90S_90N/

Supplementary Table 3: Main characteristics of the ECCO2 model.

Model number	Model name	Version	Resolution	Compartments represented	Forcings data	Availability	Reference	Website
1	CLM	2.0	1°	SMS, SWE,	CMAP + NOAA/GDAS	1979-2016	[24]	https://disc.sci.gsfc.nasa.gov/services/disc/services/grads-gds/gldas
2	MOSAIC		1°	SMS, SWE	CMAP + NOAA/GDAS	1979-2016	[24]	https://disc.sci.gsfc.nasa.gov/services/disc/services/grads-gds/gldas
3	NOAH	2.7	0.25°	SMS, SWE	CMAP + NOAA/GDAS	1979-2016	[24]	https://disc.sci.gsfc.nasa.gov/services/disc/services/grads-gds/gldas
4	VIC		1°	SMS, SWE	CMAP + NOAA/GDAS	1979-2016	[24]	https://disc.sci.gsfc.nasa.gov/services/disc/services/grads-gds/gldas
5	WGHM	2.1	0.5°	SWS, SMS, GWS, SWE, ant.	GPCC + ECMWF	2002-2012	[25,26]	https://www.uni-frankfurt.de/45218063/WaterGAP
6	WGHM	2.2	0.5°	SWS, SMS, GWS, SWE, ant.	GPCC + ECMWF	2002-2012	[27,28]	https://www.uni-frankfurt.de/45218063/WaterGAP
7	AWRA-L	V1	1°	SMS, SWE	WFDEI	1979-2012	[29,30]	http://www.wenfo.org/wald/data-software/ http://eos.csiro.au/awra/
8	AWRA	reanalysis	1°	Assimilation of GRACE	Princeton University	2003-2012	[31]	http://www.wenfo.org/wald/data-software/

Supplementary Table 4: Datasheet of the hydrological models used in this study

Processing strategy	Aswan inverted alone [Gt yr^{-1}]	Aswan, inverted with Toshka [Gt yr^{-1}]	Toshka, inverted with Aswan [Gt yr^{-1}]	Total mass inverted (Aswan+Toshka) [Gt yr^{-1}]	Correlation (TS inversion)
T120	-0.57	-0.56	-0.32	-0.88	.85
T96G300	-4.1	-2.8	-1.0	-3.8	.48
T96DDK7	-2.4	-0.56	-1.9	-2.45	.49

Supplementary Table 5: Long-term evolution of storage in Aswan lake and Toshka lake as inverted by GRACE. This is to be compared to -0.93 Gt yr^{-1} estimated by satellite altimetry

SUPPLEMENTARY REFERENCES

1. Martinez, J. M., Guyot, J. L., Filizola, N. & Sondag, F. Increase in suspended sediment discharge of the Amazon River assessed by monitoring network and satellite data. *Catena* **79,** 257–264 (2009).

2. Garzanti, E. *et al.* Mineralogical and chemical variability of fluvial sediments 2. Suspended-load silt (Ganga-Brahmaputra, Bangladesh). *Earth Planet. Sci. Lett.* **302,** 107–120 (2011).

3. Milliman, J. D. & Farnsworth, K. L. *River Discharge to the Coastal Ocean: A Global Synthesis*. *Cambridge University Press* (2011).

4. Chen, Z., Li, J., Shen, H. & Zhanghua, W. Yangtze River of China: Historical analysis of discharge variability and sediment flux. *Geomorphology* **41,** 77–91 (2001).

5. Gao, J. H. *et al.* Variations in quantity, composition and grain size of Changjiang sediment discharging into the sea in response to human activities. *Hydrol. Earth Syst. Sci.* **19,** 645–655 (2015).

6. Robinson, R. *et al.* The Irrawaddy River Sediment Flux to the Indian Ocean: The Original Nineteenth-Century Data Revisited. *J. Geol.* **115,** 629–640 (2007).

7. Ali, K. F. & De Boer, D. H. Spatial patterns and variation of suspended sediment yield in the upper Indus River basin, northern Pakistan. *J. Hydrol.* **334,** 368–387 (2007).

8. Wang, H. *et al.* Stepwise decreases of the Huanghe (Yellow River) sediment load (1950-2005): Impacts of climate change and human activities. *Glob. Planet. Change* **57,** 331–354 (2007).

9. Wang, H. *et al.* Recent changes in sediment delivery by the Huanghe (Yellow River) to the sea: Causes and environmental implications in its estuary. *J. Hydrol.* **391,** 302–313 (2010).

10. Restrepo, J. D. & Kjerfve, B. Magdalena river: Interannual variability (1975-1995) and revised water discharge and sediment load estimates. *J. Hydrol.* **235,** 137–149 (2000).

11. Nageswara Rao, K. *et al.* Palaeogeography and evolution of the Godavari delta, east coast of India during the Holocene: An example of wave-dominated and fan-delta settings. *Palaeogeogr. Palaeoclimatol. Palaeoecol.* **440,** 213–233 (2015).

12. Harris, P. T., Hughes, M. G., Baker, E. K., Dalrymple, R. W. & Keene, J. B. Sediment transport in distributary channels and its export to the pro-deltaic environment in a tidally dominated delta: Fly River, Papua New Guinea. *Cont. Shelf Res.* **24,** 2431–2454

(2004).

13. Wolanski, E., Gibbs, R. J., Spagnol, S., King, B. & Burnskill, G. Inorganic sediment budget in the mangrove-fringed Fly River Delta, Papua New Guinea. *Mangroves Salt Marshes* **2,** 85–98 (1998).

14. Laraque, A. *et al.* A comparison of the suspended and dissolved matter dynamics of two large inter-tropical rivers draining into the Atlantic Ocean: The Congo and the Orinoco. *Hydrol. Process.* **27,** 2153–2170 (2013).

15. Walling, D. E. The Changing Sediment Load of the Mekong River. *Ambio* **37,** 150–157 (2008).

16. Kummu, M. & Varis, O. Sediment-related impacts due to upstream reservoir trapping, the Lower Mekong River. *Geomorphology* **85,** 275–293 (2007).

17. Allison, M. A. *et al.* A water and sediment budget for the lower Mississippi-Atchafalaya River in flood years 2008-2010: Implications for sediment discharge to the oceans and coastal restoration in Louisiana. *J. Hydrol.* **432–433,** 84–97 (2012).

18. Menemenlis, D. *et al.* ECCO2: High resolution global ocean and sea ice data synthesis. *Mercat. Ocean Q. Newsl.* **31,** 13–21 (2008).

19. Menemenlis, D. *et al.* NASA Supercomputer Improves Prospects for Ocean Climate Research. *EOS Trans. AGU* **86,** 89–96 (2005).

20. Marshall, J., Adcroft, A., Hill, C., Perelman, L. & Heisey, C. A finite-volume, incompressible Navier Stokes model for studies of the ocean on parallel computers. *J. Geophys. Res.* **102,** 5753–5766 (1997).

21. Smith, W. H. & Sandwell, D. Global Sea Floor Topography from Satellite Altimetry and Ship Depth Soundings. *Science (80-.).* **277,** 1956–1962 (1997).

22. Gebco team. The General Bathymetric Chart of the Oceans (GEBCO). (2017). Available at: http://www.gebco.net/.

23. Zhang, J., Hibler, W., Steele, M. & Rothrock, D. A. Arctic Ice – Ocean Modeling with and without Climate Restoring. *J. Phys. Oceanogr.* **28,** 191–217 (1998).

24. Rodell, M. *et al.* The Global Land Data Assimilation System. *Bull. Am. Meteorol. Soc.* **85,** 381–394 (2004).

25. Döll, P., Kaspar, F. & Lehner, B. A global hydrological model for deriving water availability indicators: model tuning and validation. *J. Hydrol.* **270,** 105–134 (2003).

26. Hunger, M. & Döll, P. Value of river discharge data for global-scale hydrological modeling. *Hydrol. Earth Syst. Sci.* **4,** 4125–4173 (2007).

27. Müller Schmied, H. *et al.* Variations of global and continental water balance components as impacted by climate forcing uncertainty and human water use. *Hydrol. Earth Syst. Sci.* **20,** 2877–2898 (2016).

28. Döll, P., Müller Schmied, H., Schuh, C., Portmann, F. T. & Eicker, A. Global-scale assessment of groundwater depletion and related groundwater abstractions: Combining hydrological modeling with information from well observations and GRACE satellites. *Water Resour. Res.* **50,** 5698–5720 (2014).

29. van Dijk, A. *The Australian Water Resources Assessment System: Technical Report 3. Landscape Model (version 0.5) Technical Description*. *WIRADA/CSIRO Water for a Healthy Country Flagship, Canberra.* (2010).

30. van Dijk, A. I. J. M., Peña-Arancibia, J. L., Wood, E. F., Sheffield, J. & Beck, H. E. Global analysis of seasonal streamflow predictability using an ensemble prediction system and observations from 6192 small catchments worldwide. *Water Resour. Res.* **49,** 2729–2746 (2013).

31. van Dijk, A. I. J. M., Renzullo, L. J., Wada, Y. & Tregoning, P. A global water cycle reanalysis (2003-2012) merging satellite gravimetry and altimetry observations with a hydrological multi-model ensemble. *Hydrol. Earth Syst. Sci.* **18,** 2955–2973 (2014).
